# Supplementary material for: Multiplexed micronutrient, inflammation, and malarial antigenemia assessment using a plasma fractionation device
Source: PLoS One. 2022 Nov 21;17(11):e0277835. doi: 10.1371/journal.pone.0277835 (PMC9678258; doi:10.1371/journal.pone.0277835)
Supplement: Attachment — Submitted filename: PLoS PCD Rebuttal Letter.docx [file pone.0277835.s002.docx]

21^st^ October 2022

Dear Editor,

Pleased find below our responses to the Reviewer’s comments. We have also reviewed and proofread the draft with much greater rigor and are confident that we have clearly documented each of the edits suggested in the primary and secondary reviews by the Reviewer. We hope that these are sufficient to permit publication of our work and we thank you both for your inputs and patience in this process.

Best regards,

David Boyle

**Responses to the Reviewer’s comments**

Firstly, the project team would like to thank the Reviewer of this manuscript most sincerely for taking the time and effort to review our redrafting of the original manuscript. We were too hasty in trying to send back the edited document and therefore apologise for any time wasted by the Reviewer in light of a clean draft in being mistakenly labelled as in Track Changes. We have read and acted upon the Reviewer’s comments and our responses to them are below.

**General:** I am glad the authors found comments to be useful, and the responses and updated manuscript demonstrates that comments were thoughtfully considered. I did not see a track-changed version of the paper--it seemed like the original paper with updated figures and then a clean version of the updated paper was appended in the pdf I received--so comments below are based on what seemed to be the clean copy of the revision. Also, some of the issues that were pointed out in my original comments remain, so I hope that it was not a penultimate version that was inadvertently uploaded that I reviewed.

**Response:** We are very sorry for this mix up. We are not sure what happened, but we did go back and see that most of the original edits were acted upon but of course some were not and so again we apologize for any time wasted. In the newly submitted document in Track Changes, it is easier to see where all of these original edits were made in addition to our newer edits. The “Error” text issue is solved. We have now gone over the original review and added the comments from this secondary one and I believe that we have addressed everything noted by the Reviewer in both of their reviews. We now present a new document in Track Changes, with the hope that it will be much easier for the Reviewer to read our revisions in the text where they requested.

1. Minor points—
   Line 45: delete “in”

**Response:** We have deleted “in utero” and left “fetus”. See Ln 41

2. Lines 97-99: sentence starting with “A variety of simple devices….” seems out of the blue; perhaps it could be altered somewhat to bring the reader around to the idea that such devices could have potential in MN testing but have not yet been tried.

**Response:** This sentence has been significantly modified to give greater clarity for the reader. Lns – 96 - 99.

“To support this, we propose that devices developed to passively fractionate plasma from whole blood in austere settings, primarily for viral load testing of people living with HIV [19,22], could be repurposed to include serum ferritin measurement and each of the other assays included on the MN 7-plex immunoarray.”

3. Line 244: error message remains

**Response:** This is no longer there. The entire document has also been checked for “Error”

4. Lines 254, 257: CCC should be spelled out or the abbreviation defined

**Response:** We have defined the abbreviation in the text where first noted on Ln 221. We also added the full name in the first table legend to help the reader, Ln 254.

5. Real line 290 Line 274: “Reference source not found” is embedded in the table description, and Eurofins is not completely spelled out.

**Response:** We have amended this on Lns 273 to read “* Eurofins data are excluded from sTfR results.”

6. Line 280-281: contains “(Error! Reference source not found.”

**Response:** This was resolved, we took out all the internal linking to table references, etc. to remove risk of seeing the field code errors again.

7. Lines 291-302: issue with properly referring to and interpreting Figures 4A and 4B remain. Should “Figure 3A” at line 291 refer to Figure 4A? Is this intended to be figure legend or text interpretation of figures or a combination?

**Response:** We have added more text to provide greater clarity to the figure legends for Figs 4A and 4B. We have also deleted the reference to Fig 3A in the fig.4.A legend.

8. Why are Figure 4A and Figure 4B listed without explanation at lines 299 and 302?

**Response:** These were simply placeholders for where the figures were to be placed. We have deleted both of these with the assumption that the production editor will place each figure below its respective figurehead.

9. Line 308: change “and” to “which”? add “in” after “challenging to do”

**Response:** Done, see Ln 302.

10. Line 313: delete “and”

**Response:** Done, see Ln 312.

11. Line 325: this is the first that Rs is used to denote rho in the text; be consistent with terminology

**Response:** We have amended the text accordingly upon the first instance of Spearman’s correlation coefficient and have denoted either this and/or rho as Rs thereafter, in both the text and also in the figurehead for table 3 and in Table 3.

12. Line 334: plasma is spelled incorrectly

**Response:** Corrected.

13. There are assorted other minor issues with the discussion (e.g., words compressed together) that I did not detail that could be resolved with further proofreading.
14. **Response:** We apologize for a poor review of the Discussion before sharing back with the Reviewer. We have had the document entire proofread in an attempt to eliminate any small typographic or spelling errors in the discussion and the remainder of the draft. A further example of this is where Q-plex was sometimes used to describe the assay we used, specifically this is the MN 7-plex assay and so it has been changed throughout. All are in track changes and so very clear to the Reviewer and the Editor.
